# Early‐onset fetal growth restriction: A systematic review on mortality and morbidity

**DOI:** 10.1111/aogs.13702

**Published:** 2019-09-10

**Authors:** Anouk Pels, Irene M. Beune, Aleid G. van Wassenaer‐Leemhuis, Jacqueline Limpens, Wessel Ganzevoort

**Affiliations:** ^1^ Department of Obstetrics and Gynecology Amsterdam UMC University of Amsterdam Amsterdam The Netherlands; ^2^ Department of Obstetrics and Gynecology University Medical Center Groningen University of Groningen Groningen The Netherlands; ^3^ Department of Neonatology Emma Children's Hospital Amsterdam UMC University of Amsterdam Amsterdam The Netherlands; ^4^ Medical Library Amsterdam UMC University of Amsterdam Amsterdam The Netherlands

**Keywords:** estimated fetal weight, fetal growth restriction, fetal mortality, infant mortality, morbidity, neurodevelopmental impairment

## Abstract

**Introduction:**

Severe early‐onset fetal growth restriction is an obstetric condition with significant risks of perinatal mortality, major and minor neonatal morbidity, and long‐term health sequelae. The prognosis of a fetus is influenced by the extent of prematurity and fetal weight. Clinical care is individually adjusted. In literature, survival rates vary and studies often only include live‐born neonates with missing rates of antenatal death. This systematic review aims to summarize the literature on mortality and morbidity.

**Material and methods:**

A broad literature search was conducted in OVID MEDLINE from 2000 to 26 April 2019 to identify studies on fetal growth restriction and perinatal death. Studies were excluded when all included children were born before 2000 because (neonatal) health care has considerably improved since this period. Studies were included that described fetal growth restriction diagnosed before 32 weeks of gestation and antenatal mortality and neonatal mortality and/or morbidity as outcome. Quality of evidence was rated with the GRADE instrument.

**Results:**

Of the 2604 publications identified, 25 studies, reporting 2895 pregnancies, were included in the systematic review. Overall risk of bias in most studies was judged as low. The quality of evidence was generally rated as very low to moderate, except for 3 large well‐designed randomized controlled trials. When combining all data on mortality, in 355 of 2895 pregnancies (12%) the fetus died antenatally, 192 died in the neonatal period (8% of live‐born neonates) and 2347 (81% of all pregnancies) children survived. Of the neonatal morbidities recorded, respiratory distress syndrome (34% of the live‐born neonates), retinopathy of prematurity (13%) and sepsis (30%) were most common. Of 476 children that underwent neurodevelopmental assessment, 58 (12% of surviving children, 9% of all pregnancies) suffered from cognitive impairment and/or cerebral palsy.

**Conclusions:**

When combining the data of 25 included studies, survival in fetal growth restriction pregnancies, diagnosed before 32 weeks of gestation, was 81%. Neurodevelopmental impairment was assessed in a minority of surviving children. Individual prognostic counseling on the basis of these results is hampered by differences in patient and pregnancy characteristics within the included patient groups.

**Abbreviations**
EFW: estimated fetal weight
FGR: fetal growth restriction
NDI: neurodevelopmental impairment
RCT: randomized controlled trial

**Key message**

The data of 25 included studies, reporting 2895 pregnancies complicated by fetal growth restriction, diagnosed before 32 weeks of gestation indicate that overall survival was 81%. In 12% of the surviving children cognitive impairment and/or cerebral palsy was diagnosed.

## 1 INTRODUCTION

Severe early‐onset fetal growth restriction (FGR) with placental insufficiency as its mechanism^1^ is an obstetric condition that is mostly managed in tertiary‐care hospitals. By consensus, FGR is defined as onset before 32 weeks of gestation, a fetal abdominal circumference or estimated fetal weight (EFW) below the 3rd centile or absent end‐diastolic flow in the umbilical artery, or abdominal circumference or EFW below the 10th centile combined with a pulsatility index of the uterine artery above the 95th centile and/or pulsatility index of the umbilical artery above the 95th centile.^2^ This patient group needs high amounts of care and has a high likelihood of iatrogenic premature delivery, both for fetal and for secondary maternal indications, such as the development of the maternal syndrome of preeclampsia.^3^ As these FGR children are usually born very preterm, the condition carries significant risks of neonatal mortality, major and minor morbidity, and long‐term health sequelae.^4^, ^5^ These risks are not only strongly related to gestational age, but also to the extent of growth restriction. Reported survival rates vary.^3^

Counseling patients with severe early‐onset FGR about perinatal prognosis is difficult because of the uncertain influence of different prognostic variables of the condition. Furthermore, the widespread variability of existing data on survival and long‐term prognosis of the fetus makes decision‐making in this patient group even more difficult.

Overview of total mortality is often lacking in literature on this subject. For example, many studies describe the prognosis of live‐born neonates after FGR and do not take antenatal death into account. From an obstetric perspective, long‐term outcomes can only be interpreted optimally if they are presented together with the proportions of antenatal and neonatal death.^6^ The aim of this systematic review is to describe the chances of overall (antenatal and neonatal) survival, and long‐term morbidity and neurodevelopment based on the total number of fetuses at first FGR diagnosis to inform patients and obstetricians in their counseling and decision‐making.

## 2 MATERIAL AND METHODS

### 2.1 Data sources

An information specialist (JL) performed a broad search in OVID MEDLINE from 2000 to 27 April 2019. The search consisted of controlled terms, including MeSH terms, and text words for FGR and antenatal/perinatal mortality or neurodevelopment in infants with demonstrated FGR, combined with search filters to retrieve primary and secondary studies (the latter only as a check). We searched from 2000 onwards because neonatal health care has changed fundamentally in the current millennium. No further restrictions were applied. The complete search strategy is shown in the Supplementary material (Table S1). The retrieved records were imported and de‐duplicated in endnote X7. The included studies were screened for additional relevant cited or citing references.

### 2.2 Main outcomes measures

Six important research questions were identified:

1. What is, in severe early‐onset FGR, the chance of intrauterine death?
2. What is, in live‐born neonates after severe early‐onset FGR, the chance of neonatal death?
3. What is, in surviving children after severe early‐onset FGR, the chance of neurodevelopmental impairment (NDI) at or before 5 years of age in long‐term follow up?
4. What is, in surviving children after severe early‐onset FGR, the mean cognitive score at or before 5 years of age?
5. What is, in surviving children after severe early‐onset FGR, the mean motor score at or before 5 years of age?
6. What is, in surviving children after severe early‐onset FGR, the chance of cerebral palsy at or before 5 years of age?

### 2.3 Eligibility criteria

Records covering singleton pregnancies diagnosed with FGR, as defined by trialists, diagnosed before 32 weeks of gestation, were included when the antenatal and perinatal data on mortality were reported. If a study included patients diagnosed with FGR before and after 32 weeks of gestation (for example between 24 and 38 weeks of gestation) the study was only included if data on the subgroup below 32 weeks of gestation was reported separately in the publication. Because of the progress of quality of obstetric and neonatal care, only patient groups (partially) born in or after the year 2000 were included. Furthermore, only records published in English and with an available full text were included.

Records were excluded if they only described neonates born after FGR, evaluating the postnatal data, without describing the antenatal and perinatal mortality.

### 2.4 Data collection

Titles and abstracts of all search results were independently screened by 2 researchers (AP and IMB). Discrepancies were resolved by discussion with a third researcher (WG). The full text of potentially eligible studies was assessed. Relevant data were extracted from the full text by 2 researchers independently (AP and IMB) and compared for purpose of completeness and correctness.

The quality of the evidence was rated using the GRADE instrument.^7^

## 3 RESULTS

**Figure 1:**
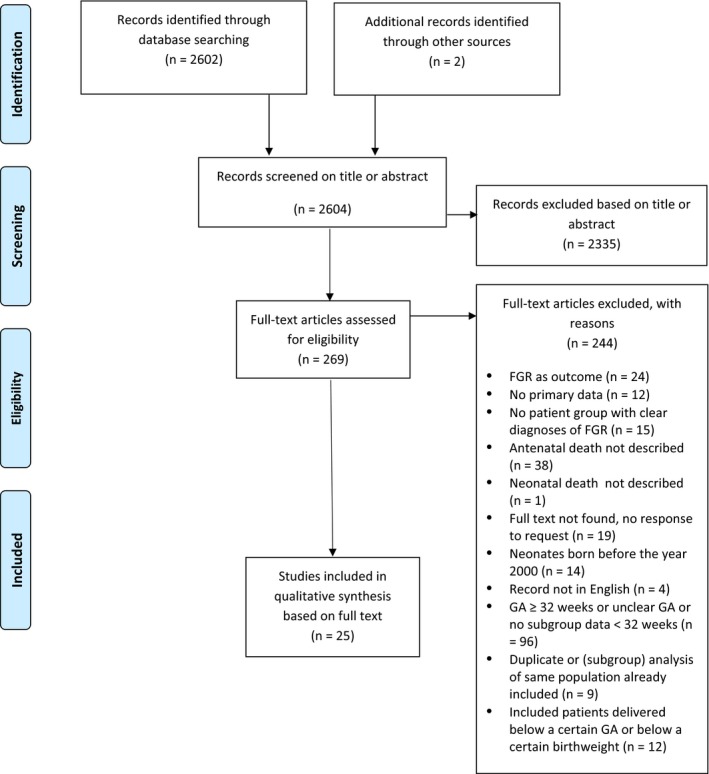
Flowchart article selection. FGR, fetal growth restriction; GA, gestational age [Color figure can be viewed at http://wileyonlinelibrary.com]

The literature search identified 2602 unique records, and 2 additional records were identified through reference and citation checks. After title and abstract screening, 269 full‐text records were assessed for eligibility; 25 studies comprising 2895 patients were included in the systematic review (Figure 1).

### 3.1 General characteristics of the studies

Table 1 summarizes the characteristics of the included studies. The number of included pregnancies varied from 8 to 503. FGR was defined differently among the included studies; some studies focused on the EFW or abdominal circumference only, whereas other studies included Doppler measurements as well (Table 1).

**Table 1:** Characteristics of included studies

	Study design	Number of patients	Definition of FGR	Gestational age at diagnosis FGR (wk + d), (mean ± SD or median [IQR])	EFW at diagnosis FGR (g) (mean ± SD or median [IQR])	Proportion of patients with preeclampsia or HELLP at diagnosis FGR
Ali et al^9^	Clinically retrospectively registered, open, parallel, randomized controlled trial	80	AC <10th centile with increased HC:AC ratio	Group 1 (n = 34) mean 30 ± 0.5 Group 2 (n = 34) mean 30 ± 0.3	Group 1 (n = 34) mean 1202 ± 72 Group 2 (n = 34) mean 1209 ± 48	0%
Ali et al^8^	Clinically registered, open, parallel, randomized clinical trial	60	AC or birthweight <10th centile	Group 1 (n = 30) mean 30 ± 0.5 Group 2 (n = 30) mean 30 ± 0.4	Group 1 (n = 30) mean 1193 ± 51 Group 2 (n = 30) mean 1216 ± 63	0%
Aoki et al^14^	Retrospective cohort study	17	<5th centile (not defined what needs to be <5th centile)	Median 25.4 (22.6‐27.7)	Median 513 (260‐741)	17/17 = 100%
Baschat et al^22^	Prospective cohort study	44	AC <5th centile and umbilical artery Doppler PI more than 2 SD above the gestational mean by local reference values	Median 25^+1^ (range 16^+4^ to 31^+6^)	Not described	Not described
Belghiti et al^23^	Retrospective cohort study	10 FGR patients with reported outcomes	<5th centile (not defined what needs to be <5th centile)	25^+0^ to 25^+6^	Not described for subgroup FGR	10/10 = 100%
Fox et al 8^17^	Retrospective case‐control study	252	EFW <25th centile	21.0 ± 1.0	Not described	Not described
Fujisaki et al^24^	Prospective, 1‐arm, interventional pilot study	14	EFW ≤5th centile	Median 25^+3^ (22^+6^ to 25^+5^)	Mean 418 ± 160	0%
Groom et al^11^	Triple‐blind, placebo‐controlled, parallel, phase II‐III trial randomized at the participant level	122	At 22^+0^ to 27^+6^ wk of gestation: AC ≤3rd centile At 28^+0^ to 29^+6^ wk of gestation: EFW <700 g	Group 1 (n = 63): Mean 24.5 ± 1.7 Group 2 (n = 59): Mean 24.8 ± 1.7	Group 1 (n = 63): Mean 479.3 ± 148.1 Group 2 (n = 59): Mean 495.7 ± 170.2	16/122 = 13.1%
Hasegawa et al^25^	Retrospective cohort study	26	<5th centile (not defined what needs to be <5th centile)	Group 1 (n = 17) median 25.3 (21.4‐29.9) Group 2 (n = 9) median 25.3 (20.4‐28.1)	Not described	Not described
Herraiz et al^26^	Observational prospective cohort study	74	EFW <3rd centile or EFW <10th centile + abnormal fetal Doppler	Group 1 (n = 37): 27.0 ± 2.8 Group 2 (n = 36): 27.9 ± 2.0	Not described	36/74 = 48.6%
Kubo et al^27^	Open label, phase 1 clinical trial	8 (<32 wk)	EFW ≤ to −1.5 SD on ultrasonography from the Japanese standard table	Median 28^+4^ (26^+0^ to 30^+5^)	Median 967 (708‐1164)	Not described
Lawin‐O'Brien et al^28^	Multicenter retrospective study of databases	245	AC ≤3rd centile for gestational age, AC calculated according to UK recommended standard and Altman and Chitty chart	Median 23^+4^ wk (range 22^+0^ to 25^+6^)	Median 353 g (range 166‐677)	81/245 = 33%
Lees et al^10^	Prospective multicenter non‐blinded management trial	503	AC below 10th centile according to local standards and abnormal umbilical artery Doppler PI above 95th centile based on local standards, irrespective of the presence of absent or reversed EDF	Mean 29^+0^ ± 11	Mean 881 ± 217 g	195/503 = 38.8%
Maged et al^29^	Prospective non‐randomized study	50	EFW <10th centile or AC <10th centile with abnormal umbilical artery Doppler indices	Group 1 (n = 25): Mean 27.4 ± 1.6 Group 2 (n = 25): Mean 28.1 ± 1.5	Not described	Not described
Petersen et al^30^	Retrospective cohort study	33 patients, with 36 pregnancies	EFW <10th centile for GA and at least 2 of the following: normal karyotype, notched uterine artery Doppler waveforms in the second trimester, placental histology changes consistent with uteroplacental insufficiency	Median 24 (range 18‐29)	Median 364 g (range 167‐496)	Not described
Rizzo et al^31^	Cohort study (unclear whether prospective or retrospective)	31	EFW <10th centile for population standard confirmed at birth	Median 26.1 wk (range 22.6‐29.1)	Not described	0/31 = 0%
Savchev et al^32^	Retrospective analysis of a prospective cohort	211 subgroup <32 wk	EFW <10th centile	Mean 28.1 ± 4.0 wk	Mean 1061 ± 494 g	74/211 = 35.1%
Sharp et al^12^	Randomized placebo‐controlled trial	135	AC or EFW <10th centile and absent or reversed EDF in the umbilical artery	Group 1 (n = 70): Median 25.1 (24.0‐27.5) Group 2 (n = 65): Median 25.6 (24.1‐27.4)	Group 1 (n = 70): Median 451 (352‐613) Group 2 (n = 65): Median 436 (326‐594)	24/135 = 17.8%
Simonazzi et al^13^	Retrospective cohort study	16	EFW and/or AC <5th centile	Median 22^+3^ (range 20^+0^ to 23^+3^)	Median 324 g (range 248‐509)	Not described
Story et al^33^	Retrospective cohort study	20	EFW <3rd centile	Median 21^+4^ (range 18^+2^ to 24^+0^)	Not described	Not described
Takahashi et al^34^	Prospective cohort study	18	<1.5 SD Japanese standard	Median 23.0 (range 18‐25)	Not described	0/18 = 0%
Temming et al^35^	Retrospective cohort study	355	EFW <10th centile using Warsof growth curves before 20^+0^ wk of gestation and Hadlock growth curves from 20^+0^ wk of gestation onward	Mean 19.5 ± 0.9	Not described	Not described
Von Dadelszen et al^36^	Case‐control study	27	AC <5th centile	Group 1 (n = 17) median 21^+1^ (19^+5^ to 23^+2^) Group 2 (n = 10) median 22^+4^ (21^+1^ to 23^+4^)	Not described	Not described
Yildirim et al^37^	Retrospective cohort study	300	EFW <10th centile	Group 1 (n = 137) median 30.8 (CI 30.3‐31.3) wk Group 2 (n = 163) 30.1 (CI 29.6‐30.6) wk	Not described	184/300 = 61.3%
Zhang‐Rutledge et al^38^	Retrospective cohort study	254	EFW ≤10th centile	Group 1 (n = 91): Average 21^+5^ Group 2 (n = 163): Average 21^+3^	Not described	Not described

Abbreviations: AC, abdominal circumference; EDF, end‐diastolic flow; EFW, estimated fetal weight; FGR, fetal growth restriction; GA, gestational age; HC, head circumference; IQR, interquartile range; PI, pulsatility index; SD, standard deviation.

Table S2A,B (see Supplementary material) shows the judgment of risk of bias of the individual studies. Two^8^, ^9^ of the 5 included randomized controlled trials (RCT)^8^, ^9^, ^10^, ^11^, ^12^ were judged as “unknown” risk of bias. This judgment was mostly based on the fact that these studies were retrospectively registered and not blinded, and that some of the baseline criteria and outcomes were not reported for pregnancies that involved neonatal death. The other RCTs and the observational studies included were generally judged as “low” risk of bias.

### 3.2 Synthesis of the results

The results on mortality are summarized in Table 2. When combining all data on mortality, of 2895 pregnancies, 355 (12.3%; range 0%‐53%) ended in an antenatal death. Of 2540 live‐born children, 1 child was lost to follow up. In all, 192 (7.6% of 2539 and 6.6% of the total of 2895 pregnancies; range 0%‐71%) neonatal deaths occurred, and 2347 (81%; range 14%‐100%) of pregnancies survived.

**Table 2:** Outcome data on mortality

	Number of patients in final analysis	GA at delivery (wk + d) (mean ± SD or median [IQR])	Birthweight (g) (mean ± SD or median [IQR])	Antenatal death	Live born	Neonatal death	Survival at discharge
Ali et al^9^	73 7/80 lost to follow up	Group 1 (n = 34) mean 36 ± 0.9 Group 2 (n = 34) mean 36 ± 0.7	Group 1 (n = 34) mean 2022 ± 25 Group 2 (n = 34) mean 2324 ± 19	0/73 = 0%	73/73 = 100%	5/73 = 6.8%	68/73 = 93.2%
Ali et al^8^	55 5/60 lost to follow up	Group 1 (n = 25) mean 36.8 ± 0.8 Group 2 (n = 20) mean 34.8 ± 0.6. (among surviving babies)	Group 1 (n = 25) mean 1854 ± 262 Group 2 (n = 20) mean 1694 ± 169 (among surviving babies)	0/55 = 0%	55/55 = 100%	10/55 = 18.2%	45/55 = 81.8%
Aoki et al^14^	17	Median 27.3 (23.7‐29.3) wk	Median 568 g (300‐764)	1/17 = 5.9%	16/17 = 94.1%	2/16 = 12.5%	14/17 = 82.4%
Baschat et al^22^	44	Median 29^+6^ (range 26^+4^ to 37^+6^) for live birth Median 26^+6^ (range 25^+1^ to 28) for stillbirth	Median 725 (range 420‐2260)	10/44 = 22.7%	34/44 = 77.3%	1/34 = 2.9%	33/44 = 75.0%
Belghiti et al^23^	10 with reported outcome	Median 26^+2^ (25^+6^ to 26^+6^)	Median 507 (429‐553)	1/10 = 10.0%	9/10 = 90.0%	6/9 = 66.7%, of which 4 intrapartum death	3/10 = 30.0%
Fox et al^17^	252	Mean 39.2 ± 2.4	Mean 2999 ± 682 g	4/252 (1.6%)	248/252 (98.4%)	2/248 = 0.8%	246/252 = 97.6%
Fujisaki et al^24^	14	Median 29^+0^ (26^+6^ to 35^+3^)	Median 604 (437‐1340)	2/14 = 14.3%	12/14 = 85.7%	1/12 = 8.3%	11/14 = 78.6%
Groom et al^11^	122	Group 1 (n = 63): Mean 31^+5^ ± 4^+4^ Group 2 (n = 59): Mean 31^+2^ ± 4^+4^	Group 1 (n = 63): 1233 ± 774 Group 2 (n = 59): 1184 ± 823	19/122 = 15.6%	103/122 = 84.4%	9/103 = 8.7%	94/122 = 77.0%
Hasegawa et al^25^	26	Group 1 (n = 17) median 28.7 (24.7‐31.7) Group 2 (n = 9) median 28.5 (26.1‐32.4)	Group 1 (n = 18) median 695 (424‐1016) g Group 2 (n = 8) median 568 (426‐654) g	1/26 = 3.8%	25/26 = 96.2%	1/25 = 4%	24/26 = 92.3%
Herraiz et al^26^	73 1/74 lost to follow up	Group 1 (FGR) mean 30.1 ± 3.2 Group 2 (FGR+PE) mean 29.4 ± 2.5	Group 1 (FGR): mean 994 ± 419 Group 2 (FGR+PE): 925 ± 308	4/73 = 5.5% (1 TOP and 3 IUFD)	69/73 = 94.5%	6/69 = 8.7%	63/73 = 86.3%
Kubo et al^27^	8	Median 37^+0^ (35^+2^ to 37^+0^)	Median 2157 (1553‐2281)	0/8 = 0%	8/8 = 100%	0/8 = 0%	8/8 = 100%
Lawin‐O'Brien et al^28^	245 79/324 lost to follow up	128 (52.2%) <28^+0^ 53 (21.6%) 28^+1^ to 32^+0^ 24 (9.8%) 32^+1^ to 36^+0^ 36 (14.7%) >36^+0^	Survived: Median 1020 g (range 435‐3420) Neonatal death: median 560 (range 313‐2550) Fetal death median 422 (range 155‐2570) Feticide/TOP median 345 (range 220‐512)	89 fetal death and 33 feticide/TOP = 122/245 = 49.8%	123/245 = 50.2%	22/123 = 17.9%	101/245 = 41.2%
Lees et al^10^	503 9/511 lost to follow up	Mean 30^+5^ ± 16	Mean 1013 ± 321 g	12/503 = 2.4%	491/503 = 97.6%	27/490 = 5.5% (1 live‐born lost to follow up)	463/503 = 92.0%
Maged et al^29^	50	Group 1 (n = 25): Mean 35.3 ± 1.8 Group 2 (n = 25): Mean 34.8 ± 1.9	Group 1 (n = 25): Mean 2067 ± 352 Group 2 (n = 25): 1733 ± 361	4/50 = 8.0%	46/50 = 92.0%	4/46 = 8.7%	42/50 = 84.0%
Petersen et al^30^	33 patients, with 36 pregnancies	IUFD: Median 25 wk (range 21‐27) Live birth: Median 27 wk (range 24‐31)	IUFD: Median 308 (range 170‐480) Live birth: Median 486 (320‐553)	19/36 = 52.8%	17/36 = 47.2%	12/17 = 70.6%	5/36 = 13.9%
Rizzo et al^31^	31	Median 28.3 wk (range 23.6‐30.4)	Median 590 g (range 312‐915)	7/31 = 22.6%	24/31 = 77.4%	3/24 = 12.5%	21/31 = 67.7%
Savchev et al^32^	211	Mean 34.6 ± 8.0 wk	Mean 1647 ± 765 g	9/211 = 4.3%	202/211 = 95.7%	6/202 = 3.0%	196/92.9%
Sharp et al^12^	135	Group 1 (n = 70): Median 28.1 (26.7‐29.7) Group 2 (n = 65): Median 28.4 (27.3‐30.1)	Group 1 (n = 70): Median 604 (496‐766) Group 2 (n = 65): Median 590 (430‐842)	43/135 = 31.9%	92/135 = 68.1%	17/92 = 18.5%	75/135 = 55.6%
Simonazzi et al^13^	16	Group 1 (n = 4) median 34 wk (30‐36) Group 2 (n = 11) median 28 wk (24‐30)	Group 1 (n = 4) median 1598 g (1100‐1750) Group 2 (n = 11) median 630 g (408‐951)	1/16 TOP = 6.25%	15/16 = 93.8%	3/15 = 20%	12/16 = 75.0%
Story et al^33^	20	Survived (n = 12): median 32^+0^ (range 27^+1^ to 39^+0^) Neonatal death (n = 2): 26 wk and 31^+5^ IUFD (n = 6): median 26 (range 24^+2^ to 27^+2^)	Survived (n = 12): median 980 (range 720‐2090) Neonatal death (n = 2): 620 and 1050 Fetal death (n = 6): median 450 (range 424‐530)	6/20 = 30%	14/20 = 70%	2/14 = 14.3%	12/20 = 60.0%
Takahashi et al^34^	18	Median 28.5 wk (26.0‐30.3)	Median 625 g (3630‐850)	5/18 = 27.8%	13/18 = 72.2%	2/13 = 15.4%	11/18 = 61.1%
Temming et al^35^	355	Mean 37.2 ± 3.4	Mean 2725 ± 763 g	9/355 (2.5%)	346/355 (97.5%)	5/346 (1.4%)	341/355 = 96.1%
Von Dadelszen et al^36^	27	Group 1 (n = 17): median 25^+6^ (23^+5^ to 29^+5^) Group 2 (n = 10): median 27^+1^ (25^+4^ to 32^+6^)	Not described	14/27 = 51.9%	13/27 = 48.1%	2/13 = 15.4%	11/27 = 40.7%
Yildirim et al^37^	300	Group 1 (n = 137): median 32.8 (95% CI 32.3‐33.3) wks Group 2 (n = 163): median 31.3 (95% CI 30.84‐31.7) wk	Group 1 (n = 137) median 1390 (95% CI 1308‐1473) g Group 2 (n = 163) median 1071 (95% CI 1011‐1131) g	58/300 = 19.3% (perinatal death)	242/300 = 80.7%	29/242 = 12.0% 9 deaths >28 d (9/242 = 3.7%)	204/300 = 68.0%
Zhang‐Rutledge et al^38^	254	Group 1 (n = 91): 37.1 Group 2 (n = 163): 38.3 (not described whether means or medians are presented)	Group 1 (n = 91): Average 2605 Group 2 (n = 163): Average 2936	4/254 = 1.6%	250/254 = 98.4%	6/250 = 2.4%	244/254 = 96.1%

Abbreviations: FGR, fetal growth restriction; GA, gestational age; IQR, interquartile range; IUFD, intrauterine fetal death; PE, preeclampsia; SD, standard deviation; TOP, termination of pregnancy.

A subset of the studies report neonatal morbidity (see Supplementary material, Table S3). When combining the data, 34% of the live‐born neonates experienced respiratory distress syndrome (2 studies, range 34%‐36%), 9.1% had bronchopulmonary dysplasia (4 studies, range 4%‐19%), 4.3% had intraventricular hemorrhage (10 studies, range 0%‐25%), 5.6% had necrotizing enterocolitis (9 studies, range 0%‐22%), 2.6% had persistent pulmonary hypertension of the newborn (2 studies, range 1.9%‐9.1%), 12.5% had retinopathy of prematurity (4 studies, range 2%‐29%) and 30% had sepsis (4 studies, range 25%‐64%). One study used a composite outcome for severe neonatal morbidity^13^ and 1 study used a composite for respiratory distress syndrome and chronic lung disease.^14^

The ages at which the neurodevelopmental outcome was assessed, the types of tests used for the assessment and the definition of NDI differed between studies. Therefore, not all studies could be included in the evidence table. From the 476 children (402 from 1 larger study, the remainder from 6 small studies) who underwent neurodevelopmental assessment (Table 3), 58 children (12%; 0%‐27%) suffered from cognitive impairment and/or cerebral palsy. Overall, cerebral palsy rates in the 7 studies were low: varying from 1% to 10%. NDI was diagnosed in 50 children (11% of surviving children assessed). Eight percent of 629 pregnancies resulted in a surviving infant with NDI. Only Lees et al,^10^ reporting 10% NDI among the assessed children, included all important domains in the definition of NDI (Bayley III score, cerebral palsy, hearing loss and visual loss).

**Table 3:** Outcome data on long‐term follow up

	Number of surviving children assessed in follow up	Age at assessment	Definition of NDI	Neurodevelopmental test used	Proportion of children with NDI
Aoki et al^14^	12/14 = 85.7%	Not described	“Handicapped” (not further explained)	Not described	3/12 = 25.0% of surviving children 3/17 = 17.6% of all pregnancies
Fujisaki et al^24^	11/11 = 100%	18 mo	“Mental retardation” was defined as developmental quotient of <70	Kyoto Scale of psychological Development 2001	3/11 = 27.3% of surviving children 3/14 = 21.4% of all pregnancies
Hasegawa et al^25^	23/23 = 100%	2 y (corrected)	Neurological complications were defined as cerebral palsy or mental retardation diagnosed by independent pediatric neurologists at corrected age of 2 y	Not described	5/23 = 21.7% of surviving children (1 cerebral palsy, 4 mental retardation) 5/26 = 19.2% of all pregnancies
Lees et al^20^	443/461 = 88%	2 y, corrected for prematurity	A cognitive Bayley III score or corrected Bayley II mental development index score of less than 85 or an estimated cognitive delay of more than 3 mo, cerebral palsy, with a GMFCS of more than 1, hearing loss needing hearing aids, or severe visual loss (legally certifiable as blind or partially sighted)	Bayley III Scales of Infant and Toddler Development or corrected Bayley II	39/402 = 9.7% of surviving children (443 with known outcome, but 402 neurodevelopmental assessed) 39/502 = 7.8% of all pregnancies Cerebral palsy 6/402 = 1.5% of surviving children Cerebral palsy 6/502 = 1.2% of all pregnancies
Petersen et al^30^	5/5 = 100%	2 y of age (corrected)	Developmental delay was defined as >1 SD below the mean	Griffiths Mental Developmental Scales	1/5 = 20.0% of surviving children 1/36 = 2.8% of all pregnancies
Simonazzi et al^13^	12/12 = 100%	Median 30 mo (24‐58 mo)	Not described	Not described	0/12 = 0% Cerebral palsy 1/12 = 8.3% of surviving children Cerebral palsy 1/16 = 6.3% of all pregnancies
Takahashi et al^34^	11/11 = 100%	Between 2 and 13 y median 6 y	Developmental quotient <70	Kyoto scale of development and the Wechsler Intelligence Scale for Children III	0/11 = 0% Cerebral palsy 1/11 = 9.1% of surviving children Cerebral palsy 1/18 = 5.6% of all pregnancies

Abbreviations: GMFCS, gross motor function classification system; NDI, neurodevelopmental impairment; SD, standard deviation.

Tables 4 and 5 present the quality of evidence for our research questions on the mortality and long‐term neurodevelopment, respectively. Our fourth and fifth research questions were not addressed in any of the included studies.

**Table 4:** Evidence table on mortality outcomes

No. of studies	Certainty assessment						Effect		Certainty	Importance
	Study design	Risk of bias	Inconsistency	Indirectness	Imprecision	Other considerations	No. of events	No. of individuals		
Antenatal death—RCT										
5	Randomized trials	Serious^a^	Serious^b^	Not serious	Not serious	None	74	888	LOW	CRUCIAL
Antenatal death—observational studies										
20	Observational studies	Not serious	Very serious^c^	Serious	Not serious	None	281	2007	VERY LOW	CRUCIAL
Neonatal death—RCT										
5	Randomized trials	Serious^a^	Serious^d^	Not serious	Not serious	None	68	813	LOW	CRUCIAL
Neonatal death—observational studies										
20	Observational studies	Not serious	Very serious^e^	Serious^f^	Not serious	None	123	1726	VERY LOW	CRUCIAL

Abbreviation: FGR, fetal growth restriction; GA, gestational age; RCT, randomized controlled trial.

a Two out of 5 randomized controlled trials were rated as unknown risk of bias.

b Rate of antenatal death varies between 0% and 31.9%.

c Rate of antenatal death varies between 0% and 52.8%.

d Rate of neonatal death varies between 5.5 and 18.5%.

e Rate of neonatal death varies between 0% and 70.6%.

f Definitions of FGR and GA at inclusion differ.

**Table 5:** Evidence table on neurodevelopmental outcomes

No. of studies	Certainty assessment						Effect		Certainty	Importance
	Study design	Risk of bias	Inconsistency	Indirectness	Imprecision	Other considerations	No. of events	No. of individuals		
Neurodevelopmental impairment at or before 5 y of age in long‐term follow up—RCT										
1	Randomized trials	Not serious	Not serious	Not serious	Not serious	None	39	402	HIGH	CRUCIAL
Neurodevelopmental impairment at or before 5 y of age in long‐term follow up—observational studies										
2	Observational studies	Not serious	Not serious	Serious^a^	Serious^b^	None	6	28	LOW	CRUCIAL
Cerebral palsy at or before 5 y of age—RCT										
1	Randomized trials	Not serious	Not serious	Not serious	Not serious	None	6	402	HIGH	IMPORTANT
Cerebral palsy at or before 5 y of age—observational studies										
2	Observational studies	Not serious	Not serious	Serious	Serious^b^	None	1	28	LOW	IMPORTANT

Abbreviation: FGR, fetal growth restriction, GA, gestational age; RCT, randomized controlled trial.

a Definitions of FGR and GA at inclusion differ.

b Small sample size of 1 study (5 children).

## 4 DISCUSSION

The aim of this systematic review was to collate evidence on the perinatal mortality, morbidity and long‐term (neuro‐)development of pregnancies complicated by early‐onset FGR. Particularly in pregnancies with fetal compromise around the limits of viability, information on fetal and neonatal prognosis could offer a guide in decision‐making for parents and obstetricians.

We found that antenatal mortality was about twice as high as neonatal mortality. Only a few studies reported on the number of children diagnosed with relevant neonatal morbidity, such as respiratory distress syndrome, bronchopulmonary dysplasia, persistent pulmonary hypertension of the newborn and retinopathy of prematurity. Also, a minority of the studies reported outcomes of long‐term follow up. Moreover, neurodevelopmental assessments were performed at different ages and different neurodevelopmental measures were used.

The strength of this systematic review is the broad literature search and the strict inclusion criteria. We excluded studies that included all their patients before 2000, as the level of (neonatal) health care was essentially different in that period. Many studies that reported long‐term follow up did not include the antenatal and/or neonatal mortality of the sample studied,^5^, ^15^ which could create selection bias and may lead to numbers on healthy survival of early‐onset severe FGR to be too optimistic. Therefore, we also predefined to exclude studies that used live birth or survival as starting criteria, as we consider it crucial to include data on all‐type mortality to allow proper conclusions about prognosis from the obstetric perspective. Severity of brain damage is not only associated with FGR, but also with perinatal/neonatal management, and survival bias was therefore taken into account.

One weakness of this systematic review is the lack of consistency in the definition of FGR in the included studies. As is highlighted in Table 1, only the minority of the included studies report in detail on the definition of FGR that was used. Studies basing the diagnosis of FGR only on growth parameters are especially at risk of having included small‐for‐gestational‐age pregnancies as well, even though the risk of including small‐for‐gestational‐age pregnancies without placental insufficiency is higher above 32 weeks of gestation compared with pregnancies below 32 weeks of gestation.^16^ In particular, the study of Fox et al^17^ included a wide range of pregnancies based on the EFW <25th centile. Due to the fact that these pregnancies were antenatally diagnosed as being complicated by FGR, despite the wide definition used to diagnose the FGR and the possible bias that this could cause, we decided to include the study in the systematic review. Exclusion of this study led to an increase in the overall mortality from 18.9% to 20.4%: in total, 351 pregnancies ended in antenatal death and 190 in neonatal death, out of 2643 pregnancies (mortalities of 13.2% and 7.2%, respectively). The gestational age and EFW at diagnosis of FGR varied between the included studies and within some of the individual studies (with wide ranges or SD), possibly representing pregnancies with variable prognosis. The variety of definitions of FGR used and the range of gestational age and/or EFW of the included pregnancies are 2 of the reasons why the quality of evidence for most outcomes was rated very low, low or moderate, because the quality of evidence was downgraded due to serious indirectness^18^ based on differences in study populations.

Another weakness is the lack of consistent information about hypertensive disorders of pregnancy as they share pathophysiology and often coincide. Interventions in the management of this syndrome may have caused bias in an unknown direction.^19^

One large well‐designed RCT^20^ provides high‐quality evidence on the mortality and morbidity outcomes and neurodevelopmental outcomes at 2 years of age.^10^ Limitations of this study are that it is a trial on patient management and some pregnancies were excluded because of fetal distress. However, the advantage of this RCT was the strict inclusion criteria of FGR and the relatively well‐organized follow up with high attrition rate.

Currently, there are no specific evidence‐based therapies for early‐onset severe FGR. In the absence of therapeutic interventions, standard management consists of intensive maternal and fetal monitoring and counseling with timed delivery. Increased fetal surveillance is performed in the period of fetal viability, so that decisions around management and timing of delivery, usually by cesarean section, can be made.^3^ Informed choices depend on data on fetal and neonatal survival and morbidity. Because of the higher antenatal mortality, we hypothesize that changing thresholds for intervention to decrease antenatal mortality may result in increased postnatal mortality or increased rates of NDIs. The aim for joint obstetric and neonatal care is to improve overall survival without impairments.

Regarding the variability of prognostic profiles between patients, a systematic review of individual patient data would be useful, to be able to individualize prognostic counseling as much as possible. We excluded studies reporting on wider ranges of gestational age. This included 2 well‐designed studies investigating long‐term neurodevelopment.^6^, ^21^ In these studies, 10 out of 34 (29%) and 14 out of 149 (10%) children, respectively, had an abnormal IQ score, of which the latter percentage is in line with the findings of this systematic review. Together with the studies included in our analyses that reported on long‐term neurodevelopment, it illustrates the need for more prospective studies starting at diagnosis of FGR and extending to early school age development of the surviving children.

## 5 CONCLUSION

In this systematic review based on 25 studies comprising 2895 pregnancies complicated by severe early‐onset FGR, we found that the overall rates of antenatal and neonatal death were 12.3% and 6.6%, respectively. Of the 476 children included in the long‐term follow up, 12.2% of the survivors (7.9% of all pregnancies) were affected by NDI and/or cerebral palsy. Data on neurodevelopment were much less reported and mostly during toddler years, and not school age. Conclusions at an individual level are hampered by the differences in study quality and prognostic characteristics. A future analysis with individual patient data might further improve individual patient counseling. Longer follow up in prospective FGR cohorts is needed to provide data on the balance between mortality and NDI.

## CONFLICT OF INTEREST

None.
