# Supplementary material for: Early‐onset fetal growth restriction: A systematic review on mortality and morbidity
Source: Acta Obstet Gynecol Scand. 2019 Sep 10;99(2):153–66. doi: 10.1111/aogs.13702 (PMC7004054; doi:10.1111/aogs.13702)
Supplement: Supplementary file 1 [file AOGS-99-153-s001.doc]

Table S1: Complete literature search

Database(s): **Ovid MEDLINE(R) and Epub Ahead of Print, In-Process & Other Non-Indexed Citations and Daily**1946 to April 26, 2019 
Search Strategy: **2019-04-27**

| **#** | **Searches** | **Results** |
| --- | --- | --- |
| 1 | Fetal Growth Retardation/ | 15528 |
| 2 | (IUGR* or FGR*).tw,kf. | 7043 |
| 3 | (grow* adj6 (retard* or restrict* or restrain* or poor or poorly or insuffic* or impair*)).tw,kf. and (exp pregnancy complications/ or (f?etus* or f?etal* or intra-uterine or intrauterine or in-utero or trimester* or pregnanc* or pregnant or gestat* or gravidit* or pre-nat* or prenat*).mp.) | 25643 |
| 4 | ((extrem* or severe) adj3 ((small adj3 gestational age) or SGA or ((f?etal or f?etus*) adj2 compromis*) or ((preterm or pre-term* or prematur* or pre-matur* or immatur*) adj (f?etus* or f?etal*)))).tw,kf. | 233 |
| 5 | (vsga or (very adj2 ((small adj2 gestational age) or SGA or ((f?etal or f?etus*) adj2 compromis*) or ((preterm or pre-term* or prematur* or pre-matur* or immatur*) adj (f?etus* or f?etal*))))).tw,kf. | 127 |
| 6 | (AREDF.tw,kf. or ((end-diastolic or flow) adj2 velocity).mp.) and (Umbilical Arteries/ or Umbilical Veins/ or (DV or UA).tw. or (ductus venos* or (umbilical adj3 arter*)).tw,kf.) [DV or UA BFV] | 2404 |
| 7 | or/1-6 [FGR 1] | 33593 |
| 8 | ((f?etal or f?etus*) adj2 compromis*).tw,kf. | 1100 |
| 9 | ((preterm or pre-term* or prematur* or pre-matur* or immatur*) adj (f?etus* or f?etal*)).tw,kf. | 978 |
| 10 | Placental Insufficiency/ | 1588 |
| 11 | (placent* adj3 (insufficien* or d*sfunct*)).tw,kf. | 2896 |
| 12 | or/8-11 [placental insufficiency, fetal compromise] | 5698 |
| 13 | Fetus/bs or Placental Insufficiency/dg or ((DV or ductus venos*) adj3 (flow or pulsatil*)).tw,kf. or ((ultrasonography, doppler/ or Ultrasonography, Doppler, Color/ or ultrasonography, prenatal/ or doppler.tw,kf.) and (Umbilical Arteries/ or blood flow velocity/ or (reverse flow or flow velocit* or pulsati* or PIV or umbilical arter* or ductus venosis or DV).tw,kf.)) | 30820 |
| 14 | 12 and 13 [FGR2 = PI, immature fetus as assesed by US/doppler] | 677 |
| 15 | 7 or 14 [FGR 1-2] | 33770 |
| 16 | Fetal Growth Retardation/mo or Delivery, Obstetric/mo or Infant, Premature, Diseases/mo | 1915 |
| 17 | fetal mortality/ or perinatal mortality/ | 2368 |
| 18 | exp fetal death/ or perinatal death/ | 29560 |
| 19 | Live Birth/ | 3132 |
| 20 | ((surviv* or death* or mortalit*) adj6 (f?etal* or f?etus* or prenatal* or pre-natal* or antenatal* or ante-natal* or perinat* or peri-nat* or uterine or intrauterin* or pregnan*)).tw,kf. | 44681 |
| 21 | (stillbirth* or still-birth*).tw,kf. | 11696 |
| 22 | ((preterm or pre-term) adj3 (surviv* or mortal* or viabilit* or death*)).tw,kf. | 2266 |
| 23 | (live birth* or live preterm birth*).tw,kf. | 22118 |
| 24 | ((f?etal* or f?etus* or prenatal* or pre-natal* or antenatal* or ante-natal* or perinat* or peri-nat* or uterine or intrauterine) adj3 outcome*).tw,kf. | 18301 |
| 25 | or/16-24 [PERINATAL mortality] | 102298 |
| 26 | ((neurodevelop* or neuro-develop* or neurocognit* or neuro-cognit* or ((motor or mental or cognitiv* or brain) adj2 develop*) or ((developmental or cognitiv*) adj2 (outcome* or index)) or cerebral palsy) and ((((umbilical adj2 arter*) or ductus venosus or DV or UA) and (doppler or veloci* or blood flow or pulsatility)) or end-diastolic flow or AREDF) and (newborn* or new* born* or neonat* or neo-nat* or postnat* or post-nat* or infant* or infancy or toddler* or graders or child or children or childhood or schoolchild* or school age* or schoolage* or puber* or juvenil* or youth or adolescence or adulthood or young adult* or adult life)).mp. [ neurodevelopment in infants with demonstrated IUGR ] | 106 |
| 27 | 25 or 26 [ PERINATAL MORTALITY OR neurodevelopment in infants with demonstrated IUGR ] | 102360 |
| 28 | 15 and 27 [FGR & PERINATAL mortality] | 7431 |
| 29 | (exp animals/ or (goat* or sheep or ovine or pig or pigs or monkey* or rabbit*).ti.) not humans/ | 4589652 |
| 30 | 28 not 29 [human studies on FGR and perinatal mortality] | 6720 |
| 31 | limit 30 to yr="2000 -Current" [human studies on FGR and perinatal mortality > 2000] | 4570 |
| 32 | meta-analysis/ or (meta analy* or metaanaly* or meta?analy*).tw,kf. or ((systematic* adj3 (review or literature or evidence or search*)) or ((summari* or review) adj3 evidence) or (search* adj12 (literature* or ((electronic or medical or biomedical) adj3 database*) or exhaustive)) or medline or pubmed or embase or (CENTRAL and cochrane) or "Central Register of Controlled Trials").tw. or (cochrane or clinical evidence or EBM).jw. [SR-Filter] | 376132 |
| 33 | 31 and 32 [sec studies on FGR & PERINATAL mortality] | 334 |
| 34 | remove duplicates from 33 [sec studies on FGR & PERINATAL mortality >2000 -deduplicated] | 323 |
| 35 | (Controlled Clinical Trial/ or Randomized Controlled Trial/ or Multicenter Study/ or Observational Study/ or comparative study/ or exp cohort studies/ or case-control studies/ or registries/ or exp databases, factual/ or datasets as topic/ or exp population surveillance/ or regression analysis/ or linear models/ or logistic models/ or "Predictive Value of Tests"/ or (cohort* or case-control* or retrospective* or prospectiv* or longitudinal* or observational or epidemiologic* or descriptive or follow-up or population-based or hospital-based or consecutive or (cumulative adj3 (incidenc* or probabil*)) or registry* or registries or ((register or registers) not (Cochrane adj3 register*)) or nationwide or nation-wide or community-wide or real-life or real-world or ((national or international) adj3 (data or databas*)) or long-term trend* or (contempor* adj3 (setting* or rate* or mortalit* or surviv* or pattern* or "use" or practice* or populat* or data)) or regression or logistic or univariate or multivariate or trial or randomi*ed or randomly allocat* or double blind*).tw,kw. or (groups or subgroup*).ab. or (trends.ti. not (review/ or review.jw,ti.)) or predict*.ti.) not ((expert or current or cochrane or clinical evidence or EBM).jw. or editorial/ or books/ or (systematic* adj3 (review or literature)).ti. or ((search* adj12 (literature* or ((electronic or medical or biomedical) adj3 database*) or exhaustive or systematic)) or medline or pubmed or embase or psychinfo or (CENTRAL and cochrane) or "Central Register of Controlled Trials").tw. or (conferenc* or congress*).hw. or Case Reports/ or ((review/ or letter/ or comment/ or meta-analysis/ or (meta analy* or metaanaly* or meta?analy*).ti,ot,kf. or (systematic* adj3 (review or literature or search*)).tw,kf.) not (Controlled Clinical Trial/ or Randomized Controlled Trial/ or Multicenter Study/ or Observational Study/ or comparative study/ or exp cohort studies/ or case-control studies/ or Databases, Factual/ or medical record*.hw.))) | 6667460 |
| **36** | **31 and 35 [human primary studies on FGR & PERINATAL mortality >2000 ]** | **2603** |
| **37** | **remove duplicates from 36 [human primary studies on FGR & PERINATAL mortality >2000 -deduplicated ]** | **2602** |

Table S2A: Risk of bias of included randomized controlled trials

|  | Allocation concealment/mode of randomisation | Blinding | Loss to follow-up | Selective outcome reporting bias | Other limitations |
| --- | --- | --- | --- | --- | --- |
| Ali, 2017 | Low | Unknown | Unknown | Unknown | Unknown |
| Ali, 2018 | Low | Unknown | Unknown | Unknown | Unknown |
| Groom, 2019 | Low | Low | Low | Low | Low |
| Lees, 2015 | Low | Unknown | Low (primary analysis) / Unknown (long term follow-up) | Low | Low |
| Sharp, 2018 | Low | Low | Low | Low | Low |

Table S2B: Risk of bias of included observational studies

|  | Development and application of appropriate eligibility criteria | Flawed measurement of both exposure and outcome | Confounding | Incomplete follow-up | Other limitations |
| --- | --- | --- | --- | --- | --- |
| Aoki, 2014 | High | Low | Low | Low | Unknown |
| Baschat, 2001 | Low | Low | Low | Low | Low |
| Belghiti, 2011 | High | Low | Low | Low | Low |
| Fox, 2008 | Low | Low | Low | Unknown | Low |
| Fujisaki, 2016 | Low | Low | Low | Low | Unknown |
| Hasegawa, 2015 | Low | Unknown | Low | Low | High |
| Herraiz, 2017 | Low | Low | Unknown | Low | Low |
| Kubo, 2017 | High | High | Low | Low | High |
| Lawin-O’Brien, 2016 | Low | Unknown | Low | Unknown | Low |
| Maged, 2018 | Low | Low | Unknown | Low | Unknown |
| Petersen, 2009 | Low | Low | Low | Low | Low |
| Rizzo, 2008 | Low | Low | Low | Low | Unknown |
| Savchev, 2014 | Low | Low | Low | Low | Low |
| Simonazzi, 2013 | Low | Unknown | Low | Low | Low |
| Story, 2015 | Low | Low | Low | Low | Low |
| Takahashi, 2014 | Unknown | Low | Low | Low | Unknown |
| Temming, 2017 | Unknown | Low | Low | Low | Low |
| Von Dadelszen, 2011 | Low | Unknown | Low | Low | Low |
| Yildirim, 2008 | Low | Low | High | Unknown | Unknown |
| Zhang-Rutledge, 2018 | Unknown | Low | Low | Low | Unknown |

Table S3: Outcome data on neonatal morbidity

|  | **RDS** | **BPD** | **IVH** | **NEC** | **PPHN** | **ROP** | **Sepsis** |
| --- | --- | --- | --- | --- | --- | --- | --- |
| Aoki, 2014 | 14 / 16 = 87.5% composite neonatal morbidity described as at least one of the following: RDS, chronic lung disease (CLD) | 14 / 16 = 87.5% composite neonatal morbidity described as at least one of the following: RDS, chronic lung disease (CLD) | 0 / 16 = 0% | 0 / 16 = 0% | Not described | Not described | Not described |
| Groom, 2019 | Not described | 20 / 103 = 19.4% | 0 / 103 = 0% (Grade 3 or 4) | 1 / 103 = 1.0% (NEC requiring surgery) | 2 / 103 = 1.9% | 2 / 103 = 1.9% (ROP ≥ Grade 3 requiring treatment) | Not described |
| Hasegawa, 2015 | 9 / 25 = 36.0% | Not described | 2 / 25 = 8% | 0 / 25 = 0% | Not described | Not described | Not described |
| Herraiz, 2017 | Not described | 4 / 63 = 6.3% | 0 / 63 = 0% | 6 / 63 = 9.5% | Not described | 12 / 63 = 19.0% | 27 / 63 = 42.9% |
| Lees, 2013 | Not described | 49 / 490 = 10.0%  (> 36 weeks) | 12 / 490 = 2.4%  (GMH Grade 3 or 4) | 16 / 490 = 3.3% (Pneumatosis and perforation combined) | Not described | Not described | Total: 154 / 490 = 31.4%. Proven 87 / 490 = 18%. Clinical suspected: 67 / 490 = 14%. |
| Petersen, 2009 | Not described | Not described | 3 / 14 = 21.4% | 2 / 14 = 14.3% | Not described | 4 / 14 = 28.6% | 8 / 14 = 64.3% |
| Rizzo, 2008 | Not described | Not described | 6 / 24 = 25.0% (grade 3 or 4) | Not described | Not described | Not described | Not described |
| Sharp, 2018 | Not described | Not described | 21 / 72 = 29.2% | 20 / 92 = 21.7% | Not described | 16 / 92 = 17.4% | Not described |
| Simonazzi, 2013 | Composite severe neonatal morbidity (at least one of the following: BPD, NEC, PVL, IVH grade >2, ROP): 6 / 15 = 40% | | | | | | |
| Takahasihi, 2014 | Not described | Not described | Not described | Not described | 1 / 11 = 9.1% (of surviving children) | Not described | Not described |
| Temming, 2017 | Not described | Not described | 1 / 346 (0.3%) | 5 / 346 (1.4%) | Not described | Not described | Not described |
| Yildirim, 2008 | 81 / 242 = 33.5% | 9 / 242 = 3.7% | 15 / 242 = 6.2% | 28 / 242 = 11.6% | Not described | Not described | 60 / 242 = 24.8% |

RDS = Respiratory Distress Syndrome; BPD = Bronchopulmonary Dysplasia; IVH = Intraventricular Hemorrhage; NEC = Necrotising Enterocolitis; ROP = Retinopathy Of Prematurity; PVL = Periventricular Leukomalacia

References

1. Ali MK, Amin ME, Amin AF, Abd El Aal DEM. Evaluation of the effectiveness of low-dose aspirin and omega 3 in treatment of asymmetrically intrauterine growth restriction: A randomized clinical trial. Eur J Obstet Gynecol Reprod Biol. 2017;210:231-5.

2. Ali MK, Abbas AM, Yosef AH, Bahloul M. The effect of low-dose aspirin on fetal weight of idiopathic asymmetrically intrauterine growth restricted fetuses with abnormal umbilical artery Doppler indices: a randomized clinical trial. J Matern Fetal Neonatal Med. 2018;31(19):2611-6.

3. Groom KM, McCowan LM, Mackay LK, Lee AC, Gardener G, Unterscheider J, et al. STRIDER NZAus: a multicentre randomised controlled trial of sildenafil therapy in early-onset fetal growth restriction. BJOG. 2019.

4. Lees CC, Marlow N, van Wassenaer-Leemhuis A, Arabin B, Bilardo CM, Brezinka C, et al. 2 year neurodevelopmental and intermediate perinatal outcomes in infants with very preterm fetal growth restriction (TRUFFLE): a randomised trial. Lancet. 2015;385(9983):2162-72.

5. Sharp A, Cornforth C, Jackson R, Harrold J, Turner MA, Kenny LC, et al. Maternal sildenafil for severe fetal growth restriction (STRIDER): a multicentre, randomised, placebo-controlled, double-blind trial. The Lancet Child & adolescent health. 2018;2(2):93-102.

6. Aoki S, Toma R, Kurasawa K, Okuda M, Takahashi T, Hirahara F. Expectant management of severe preeclampsia with severe fetal growth restriction in the second trimester. Pregnancy hypertension. 2014;4(1):81-6.

7. Baschat AA, Gembruch U, Harman CR. The sequence of changes in Doppler and biophysical parameters as severe fetal growth restriction worsens. Ultrasound Obstet Gynecol. 2001;18(6):571-7.

8. Belghiti J, Kayem G, Tsatsaris V, Goffinet F, Sibai BM, Haddad B. Benefits and risks of expectant management of severe preeclampsia at less than 26 weeks gestation: the impact of gestational age and severe fetal growth restriction. American journal of obstetrics and gynecology. 2011;205(5):465 e1-6.

9. Fox NS, Huang M, Chasen ST. Second-trimester fetal growth and the risk of poor obstetric and neonatal outcomes. Ultrasound Obstet Gynecol. 2008;32(1):61-5.

10. Fujisaki M, Furuta K, Ohhashi M, Furukawa S, Kodama Y, Kawagoe Y, et al. Antithrombin improves the maternal and neonatal outcomes but not the angiogenic factors in extremely growth-restricted fetuses at <28 weeks of gestation. J Perinat Med. 2017;45(7):837-42.

11. Hasegawa Y, Aoki S, Kurasawa K, Takahashi T, Hirahara F. Association of biparietal diameter growth rate with neurodevelopment in infants with fetal growth restriction. Taiwan J Obstet Gynecol. 2015;54(4):371-5.

12. Herraiz I, Quezada MS, Rodriguez-Calvo J, Gomez-Montes E, Villalain C, Galindo A. Longitudinal changing values of the sFlt-1/PlGF ratio in singleton pregnancies with early-onset fetal growth restriction. Ultrasound Obstet Gynecol. 2017.

13. Kubo M, Tanaka H, Maki S, Nii M, Murabayashi N, Osato K, et al. Safety and dose-finding trial of tadalafil administered for fetal growth restriction: A phase-1 clinical study. J Obstet Gynaecol Res. 2017;43(7):1159-68.

14. Lawin-O'Brien AR, Dall'Asta A, Knight C, Sankaran S, Scala C, Khalil A, et al. Short-term outcome of periviable small-for-gestational-age babies: is our counseling up to date? Ultrasound Obstet Gynecol. 2016;48(5):636-41.

15. Maged M, Wageh A, Shams M, Elmetwally A. Use of sildenafil citrate in cases of intrauterine growth restriction (IUGR); a prospective trial. Taiwan J Obstet Gynecol. 2018;57(4):483-6.

16. Petersen SG, Wong SF, Urs P, Gray PH, Gardener GJ. Early onset, severe fetal growth restriction with absent or reversed end-diastolic flow velocity waveform in the umbilical artery: perinatal and long-term outcomes. The Australian & New Zealand journal of obstetrics & gynaecology. 2009;49(1):45-51.

17. Rizzo G, Capponi A, Vendola M, Pietrolucci ME, Arduini D. Relationship between aortic isthmus and ductus venosus velocity waveforms in severe growth restricted fetuses. Prenatal diagnosis. 2008;28(11):1042-7.

18. Savchev S, Figueras F, Sanz-Cortes M, Cruz-Lemini M, Triunfo S, Botet F, et al. Evaluation of an optimal gestational age cut-off for the definition of early- and late-onset fetal growth restriction. Fetal diagnosis and therapy. 2014;36(2):99-105.

19. Simonazzi G, Curti A, Cattani L, Rizzo N, Pilu G. Outcome of severe placental insufficiency with abnormal umbilical artery Doppler prior to fetal viability. BJOG. 2013;120(6):754-7.

20. Story L, Sankaran S, Mullins E, Tan S, Russell G, Kumar S, et al. Survival of pregnancies with small for gestational age detected before 24 weeks gestation. Eur J Obstet Gynecol Reprod Biol. 2015;188:100-3.

21. Takahashi Y, Iwagaki S, Chiaki R, Iwasa T, Takenaka M, Kawabata I, et al. Amnioinfusion before 26 weeks' gestation for severe fetal growth restriction with oligohydramnios: preliminary pilot study. J Obstet Gynaecol Res. 2014;40(3):677-85.

22. Temming LA, Dicke JM, Stout MJ, Rampersad RM, Macones GA, Tuuli MG, et al. Early Second-Trimester Fetal Growth Restriction and Adverse Perinatal Outcomes. Obstet Gynecol. 2017;130(4):865-9.

23. von Dadelszen P, Dwinnell S, Magee LA, Carleton BC, Gruslin A, Lee B, et al. Sildenafil citrate therapy for severe early-onset intrauterine growth restriction. BJOG. 2011;118(5):624-8.

24. Yildirim G, Turhan E, Aslan H, Gungorduk K, Guven H, Idem O, et al. Perinatal and neonatal outcomes of growth restricted fetuses with positive end diastolic and absent or reversed umbilical artery doppler waveforms. Saudi medical journal. 2008;29(3):403-8.

25. Zhang-Rutledge K, Mack LM, Mastrobattista JM, Gandhi M. Significance and Outcomes of Fetal Growth Restriction Below the 5th Percentile Compared to the 5th to 10th Percentiles on Midgestation Growth Ultrasonography. Journal of ultrasound in medicine : official journal of the American Institute of Ultrasound in Medicine. 2018;37(9):2243-9.

26. Lees C, Marlow N, Arabin B, Bilardo CM, Brezinka C, Derks JB, et al. Perinatal morbidity and mortality in early-onset fetal growth restriction: cohort outcomes of the trial of randomized umbilical and fetal flow in Europe (TRUFFLE). Ultrasound Obstet Gynecol. 2013;42(4):400-8.
